# Don't Sweat It: Cannabinoid CB1 Receptors Reduce Sweating in a Mouse Model

**DOI:** 10.1096/fj.202601143R

**Published:** 2026-06-13

**Authors:** Natalia Murataeva, Joseph Youkilis, Yogith Rao, Alex Straiker

**Affiliations:** ^1^ Department of Psychological and Brain Sciences Indiana University Bloomington Indiana USA; ^2^ Gill Institute for Neuroscience Indiana University Bloomington Indiana USA

**Keywords:** cannabinoid, CB1, exocrine, hyperhidrosis, sweating

## Abstract

Numerous exocrine glands play key physiological roles in the body that include tearing, salivation, and lactation, as well as the control of body temperature via sweating. Malfunction of sweat glands can be deeply problematic or—in the case of anhidrosis—life‐threatening. The prevalence of sweating disorders is high, affecting millions. The few available therapies are generally of limited effectiveness. Several lines of evidence point to regulation of sweating by the cannabinoid signaling system, an arrangement that would mirror cannabinoid regulation of tearing and salivation. Mice sweat in their paws via glands that closely resemble human eccrine sweat glands, including regulation by muscarinic signaling and by temperature. We applied a galvanic skin response‐based assay to investigate cannabinoid regulation of sweating in awake, unanesthetized mice. The muscarinic agonist pilocarpine increased conductance while the antagonist glycopyrrolate reduced conductance, validating the model as a measure of sweating. The cannabinoid receptor agonist CP55940 substantially reduced conductance in wild‐type and CB2 but not CB1 receptor knockout mice. The phytocannabinoid tetrahydrocannabinol (THC) also reduced conductance, while the non‐psychoactive cannabidiol (CBD) did not. Using immunohistochemistry, we detected CB1 receptors in periglandular cholinergic axons, the anandamide‐synthesizing enzyme NAPE‐PLD in myoepithelial cells, and the anandamide metabolizing enzyme FAAH in acinar cells. This indicates that a local CB1/anandamide‐based circuit is present in mouse walking pads. In summary, we employed a novel galvanic skin response‐based assay to determine that cannabinoid CB1 receptors reduce sweating in a mouse model. This may point to a previously unappreciated effect on sweating in cannabis users.

## Graphical abstract

A new galvanic skin response‐based approach offers a simple method for measuring sweating in the paws of mice. This method is used to demonstrate that cannabinoid CB1 receptors reduce baseline sweating in mice, likely via inhibition of sympathetic acetylcholine release via axonal CB1 receptors.

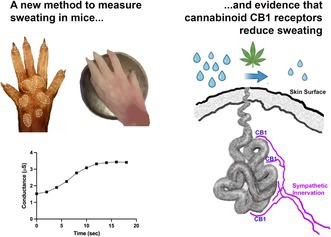

## 1 Introduction

Cannabis users' complaints of dry mouth and eyes suggested that the cannabinoid signaling system negatively regulates both tearing and salivation. We have demonstrated that cannabinoid CB1 receptors regulate the lacrimal [1], submandibular [2], and parotid [3] glands that contribute to tearing as well as basal and stimulated salivation. Lacrimal and salivary glands are part of a larger family of several dozen exocrine glands that perform important, arguably essential, functions in the body. These glands generally possess ducts to secrete fluids out of the body.

Humans are one of a small number of species (chiefly apes, monkeys, and horses) that rely heavily on sweating to regulate their body temperature [4]. Humans come equipped with several kinds of sweat glands (eccrine, apocrine, and apoeccrine) [5], but it is the eccrine sweat glands that are essential for maintaining body temperature. Malfunction of sweat glands can be deeply problematic in excess (aka hyperhidrosis) [6], or even life‐threatening in the case of anhidrosis (e.g., Fabry disease [7]). The prevalence of sweating‐related disorders is high, likely affecting tens of millions in industrialized nations [6], especially with age [8]. Few therapies are available (e.g., Botox [9]) and generally of limited effectiveness [10].

The homology of function that we have seen for cannabinoid regulation of three distinct exocrine glands raises an intriguing question: Is it possible that endogenous cannabinoids globally regulate exocrine gland function? The cannabinoid signaling system consists of several G protein‐coupled cannabinoid receptors (chiefly CB1 [11] and CB2 [12]), endogenous lipid messengers [13], and the enzymatic machinery to synthesize and metabolize these messengers [14]. CB1 receptors are implicated in the psychoactive effects of cannabis and are found both in the CNS and throughout the body. CB2 receptors, by contrast, are prominent in the immune system [12] though their expression has been reported in other tissues [15]. Reduced sweating in cannabis users has been reported anecdotally, and a recent case study reports promising results of cannabis in a patient suffering from hyperhidrosis [16]. Excessive sweating and salivation are among the reported symptoms of cannabis withdrawal in humans [17].

While mice rely on various means to thermoregulate [18], they do have eccrine sweat glands in their paws. Few studies have examined sweating in mice despite their considerable value as a research model. Studies using mice generally employ an iodine‐starch‐based colorimetric approach (e.g., [19, 20]) that either requires anesthesia or prolonged restraint. Researchers often rely on muscarinic stimulation by pilocarpine to elicit a clear sweat response. Since pilocarpine activates muscarinic (likely M_3_ [21]) receptors on the target gland, pilocarpine stimulation precludes studies of axonal release even though sweating is largely under neuronal control [22]. Since the favored model of cannabinoid action on tearing and salivation is inhibition of acetylcholine release that is upstream of muscarinic receptors [2], we explored alternative methods, ultimately adapting galvanic skin response sensors to the mouse. The method we describe is rapid, repeatable, and can be applied to awake unanesthetized mice. It is also sufficiently sensitive to measure both increases and decreases in conductance. We describe the method and its application to the study of cannabinoid regulation of sweating in mice as detailed below.

## 2 Methods

### 2.1 Animals

Mice were housed in standard ventilated caging (cage floor dimensions: 33.5 × 18 cm, depth 14 cm), with corn cob‐based bedding (Bed‐o'combs laboratory animal bedding, The Andersons, Maumee, OH, USA). Mice were group‐housed 3–4 mice per cage and were fed Inotiv Teklad 2918 irradiated rodent diet ad libitum. For most experiments, adult mice (age 2–5 months) were tested. The exception was a study of the age effects on sweating, where mice as old as eight months were tested. For key experiments, mice of both sexes were used. In particular, the CP55940 test was done separately in male and female mice since we have observed sex differences in CB1 regulation of tearing [23]. Mice on a CD1 strain background were used since we have found that, in contrast to many commonly used strains, these have an intact circadian rhythm of exocrine tearing [23] that may be relevant here. CB1 and CB2 receptor knockout mice were also on this background. Mice were kindly provided by the laboratory of Dr. Ken Mackie (Indiana University) from the same animal facility. Because mice are nocturnal, we tested mice during their active phase by maintaining them on a reverse light cycle (09:00–21:00). All animal care and experimental procedures used in this study were approved by the Institutional Animal Care and Use Committee of Indiana University (protocol# 24–030, approval date 9/3/2024) and conformed to the Guidelines of the National Institutes of Health on the Care and Use of Laboratory Animals. Experiments complied with ARRIVE guidelines.

### 2.2 Method of Measuring Galvanic Skin Responses in Mice

**FIGURE 1:**
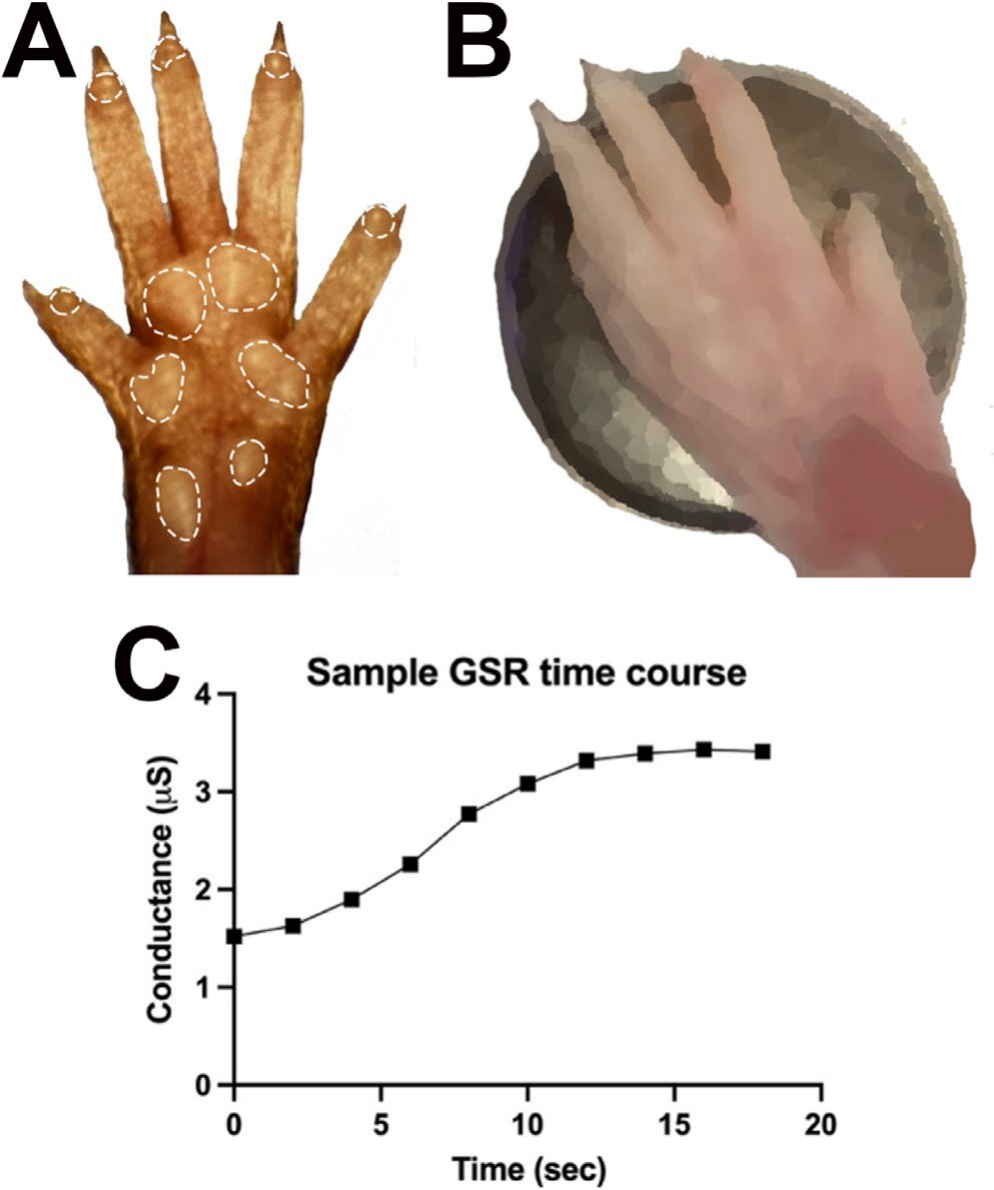
Using the galvanic skin response test in mice. (A) The figure shows the underside of the hind paw of a mouse. The main sweat gland release sites are outlined. (B) The panel shows the approximate positioning of the paw on the sensor. (C) Sample time course of conductance from the sensor.

Most of the sweat that is produced in the paw derives from the walking pads (e.g., [24]), 6 protrusions from the paw surface that make direct contact with the ground when walking (Figure 1A). This arrangement is well‐suited to the use of a galvanic skin response sensor since sweat that is produced will directly contact the sensor. We adapted Neulog galvanic skin response (GSR) sensors (https://neulog.com/gsr/) for use with mice. This system includes free software available from the manufacturer and includes two sensors that would be attached to either of two adjoining fingers in human subjects. Each sensor is a convex, rounded metal disc the size of a dime. We found that this system was readily adaptable to the hind paws of mice, provided that the mice were accustomed to handling. Each sensor is pressed to a hind paw, yielding conductance measurements in microSiemens (μS). One experimenter scruffs the mouse and presents the hind paws to a second experimenter, who gently presses each hind paw to a separate sensor. The scruffing experimenter also dabs away excess urine if needed with a tissue; both experimenters pay close attention to urine on gloves since this can confound the readings. Consistent and stable positioning of the paw is important for obtaining a reproducible measurement, but since the six central walking pads are the primary source of sweat secretion (Figure 1A), the positioning of these is readily accomplished. Paws are positioned such that the toes are splayed with toenails just off the sensor (Figure 1B).

Rear paws are tested because they are larger and more readily accessible. As noted, regular handling of the mice contributes positively to measurements. We find that once the sensors are positioned, the mice generally rest in place. Stable readings are obtained in 15–30 s, as shown in the sample time course in Figure 1C. The reading is taken at the plateau or at 30 s, whichever comes first, though in practice, very few readings (< 5%) have not reached a plateau at 30 s. The sensors are cleaned with 70% ethanol and dried before each use. One practical consideration is the state of bedding, since we find that mice from cages that are heavily soiled are more likely to yield erratic readings. This is likely due to lipids that adhere to the paws.

### 2.3 Exclusion Criteria

Mice that struggle continuously during the test yield unreliable measurements. As a rule, a second attempt is made after several minutes, but if the mouse again struggles, it is excluded from measurements. This occurs in < 5% of measurements.

For topical application, we applied 10 μL of glycopyrrolate in 100% ethanol to each hind paw. The galvanic skin response was retested after 60 min.

### 2.4 Measurement of Body Temperature

In one experiment, body temperature was measured before and after drug treatment. The tip of a rectal temperature probe (RET‐3, Kent Scientific, Torrington, CT) was dipped in Vaseline, after which the probe was inserted into the rectum of an awake, unanesthetized mouse until a stable temperature reading was obtained (as a rule, < 15 s).

### 2.5 Immunohistochemistry

For immunohistochemistry, mouse paws were fixed in 4% paraformaldehyde for ~1 h at 4°C, then placed sequentially in 10% and 30% sucrose in PBS overnight. The paw tissue was then suspended in OCT (Thermo Fisher Scientific, Waltham, MA) freezing compound in a 15 mL transparent plastic test tube, to allow precise orientation in 3‐dimensional space. The tube was then promptly submerged in cold (−80°C) ethanol to rapidly freeze the sample. Frozen paw sections (~35 μm) were collected on a Leica cryostat (Leica Microsystems, Wetzlar, Germany) and mounted on Superfrost Plus slides (Thermo Fisher). Slides were blocked with BSA (in PBS, Triton‐X, 0.3%), followed by treatment with primary antibodies (in PBS, Triton‐X, 0.3%) for 1–two days at 4°C. See Table 1 for the list of primary antibodies. Secondary antibodies were incubated for ~4 h at room temperature after washing off the primary antibody. Appropriate secondary antibodies were labeled with Alexa488, Alexa594, or Alexa647 (Thermo Fisher). Prelabeled phalloidin was included with the secondary antibodies. Slides were mounted with mounting media containing 4′,6‐diamidine‐2′‐phenylindole dihydrochloride (DAPI) to permit visualization of nuclei (Fluoromount, Sigma‐Aldrich, St. Louis, MO). Images were acquired with a Leica TCS SP8 (Leica Microsystems). Images were processed using FIJI (available at https://imagej.net/Fiji/downloads) and/or Photoshop (Adobe Inc., San Jose, CA) software. Images were modified only in terms of brightness and contrast.

**TABLE 1:** Primary antibodies used.

Target	Host	Source	Cat#	Lot#	RRID	Conc.
FAAH	Rabbit	Synaptic systems	469 003	NA	RRID:AB_2924950	1:300
CB1	Guinea pig	Frontier institute	CB1‐GP‐Af530	NA	RRID:AB_2571592	1:300
NAPE‐PLD	Guinea pig	Frontier institute	NAPE‐PLD‐GP‐Af720	NA	RRID:AB_2571806	1:300
Choline acetyl transferase	Goat	Millipore	AB144P	2 211 015	RRID:AB_2079751	1:100
Phalloidin594	N/A	ThermoFisher	A12379	1 749 905	N/A	1:500

### 2.6 Drugs

CP55940, URB597, JWH018, and pilocarpine were purchased from Cayman Chemical (Ann Arbor, MI). Glycopyrrolate was purchased from Sigma‐Aldrich (St. Louis, MO). The CB1 receptor antagonist SR141716 was purchased from Biorbyt (Durham, NC). THC and CBD were obtained through the NIDA drug supply program ADL‐19496‐11.

### 2.7 Quantification and Statistical Analysis

GraphPad Prism version 10 (La Jolla, CA) software was used for the statistical analysis. Data are presented as means ± SEM. As a rule, this study employed paired *t*‐tests comparing drug effects to same‐animal baselines. The statistical details of the experiments can be found in the results section. Differences were considered significant at *p* ≤ 0.05.

## 3 Results

### 3.1 The Galvanic Skin Response as a Measure of Sweating in Mouse Paws

**FIGURE 2:**
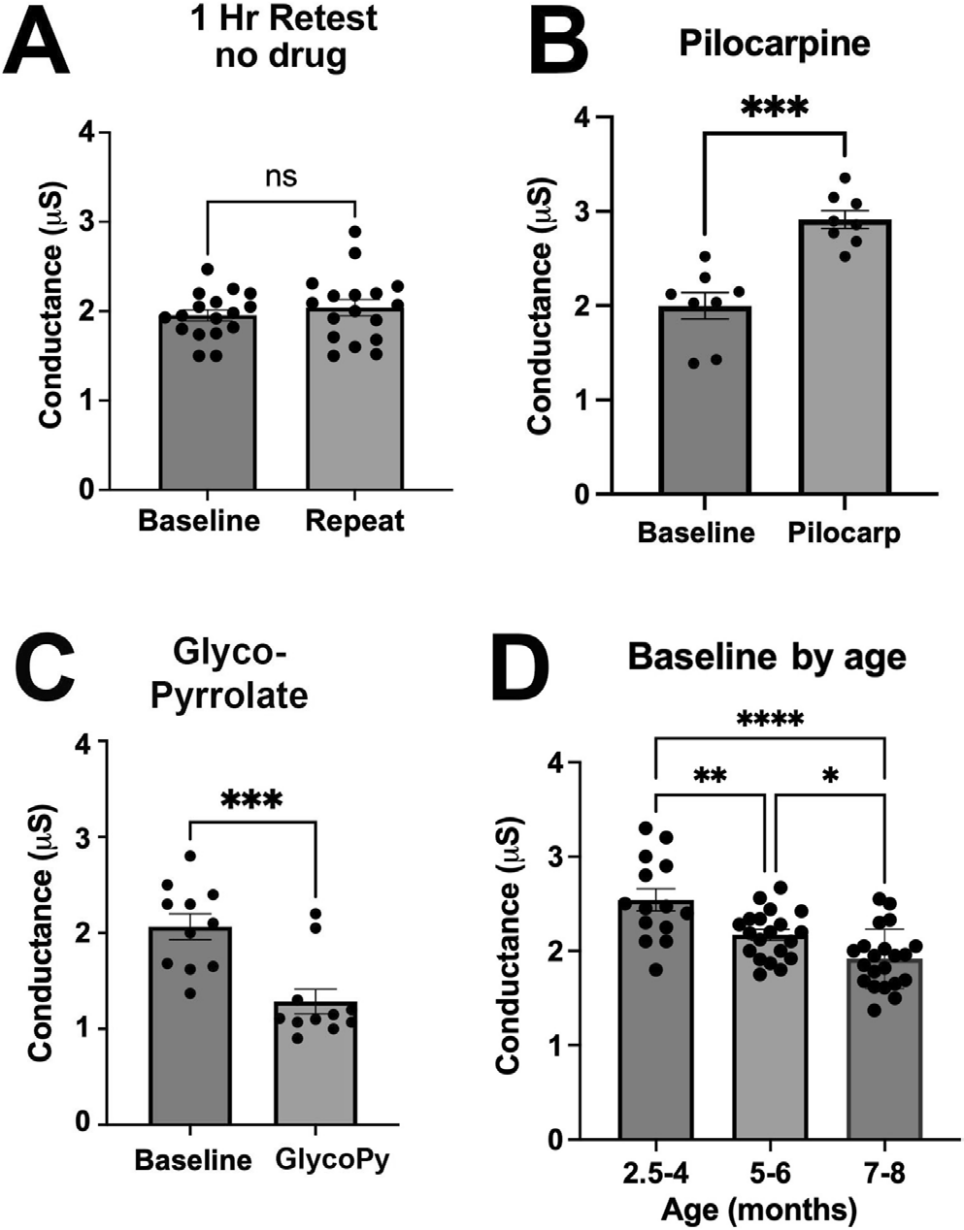
Validation of the galvanic skin response method in mice. (A) Responses are consistent over time, in this case, after one hour. (B) Muscarinic agonist pilocarpine (1 mg/kg, SC) produces the expected increase in galvanic skin response. (C) Muscarinic antagonist glycopyrrolate (2%, topical) yields the expected decrease in galvanic skin response. (D) Baselines show expected declines with age. (A–C) NS, *p* = 0.35 by paired *t*‐test against own baseline, *n* = 17; ***p = 0.0001, *n* = 8; ***p = 0.0002, *n* = 11; (D) 1‐way ANOVA with Tukey post hoc test, ****p < 0.0001; *p = 0.046; **p = 0.006; *n* = 14, 20, 21.

We first tested whether the galvanic skin response (GSR) method was consistent over time and whether the method detects an induced increase in sweating. We obtained baseline readings, then repeated measurements in the same mice after an hour. As shown in Figure 2A, the average value remained consistent in a mixture of female and male mice (Figure 2A, Baseline conductance (μS ± SEM): 1.95 ± 0.10; conductance after 1 h: 2.04 ± 0.13, *n* = 17; ns by paired *t*‐test *p* = 0.035, *t* = 0.97, df = 16). As a negative control, we also tested whether any hairless region would serve as a conductor in this assay. We found that baseline conductance was negligible when probes were tested against the ears (data not shown, conductance (μS ± SEM): 0.005 ± 0.0006; *n* = 3).

We next tested pilocarpine, a parasympathomimetic muscarinic agonist that increases sweating in mice [25]. We obtained baseline values, then after 30 min, we injected pilocarpine (1 mg/kg, subcutaneously). Ten minutes after the pilocarpine injection, we retested the GSRs, finding that these were now elevated (Figure 2B, *p* = 0.0001 by paired *t*‐test, *t* = 7.7, df = 7, *n* = 8). Pilocarpine was tested in a mixed group of male and female mice. We also tested a muscarinic antagonist, glycopyrrolate. Glycopyrrolate was prepared as a 2% solution in ethanol, then applied topically (10 μL) to each hind paw. We obtained baseline values and a second measurement 60 min after treatment, finding that values declined after glycopyrrolate treatment (Figure 2C, *p* = 0.0002 by paired *t*‐test, *t* = 5.7, df = 10, *n* = 11).

We additionally tested whether the assay could resolve the known age‐related decline in sweating seen in humans [8] as well as mice [26]. We tested female mice grouped by age (2.5–4, 5–6, and 7–8 months), finding that baseline conductance decreased progressively with age (Figure 2D; One‐way ANOVA (F (2, 52) = 14.88, *p* < 0.0001). Tukey's multiple comparisons test revealed significant differences between all three groups: 2.5–4 vs. 5–6 months (mean difference 0.37 μS, *p* = 0.006), 2.5–4 vs. 7–8 months (*p* < 0.0001), and 5–6 vs. 7–8 months (*p* = 0.046), *n* = 14, 20, 21). The assay therefore detected the expected age‐dependent decline in baseline sweating, consistent with prior reports.

### 3.2 Effects of Cannabinoid Receptor Activation on the Galvanic Skin Response in Mouse Paws

**FIGURE 3:**
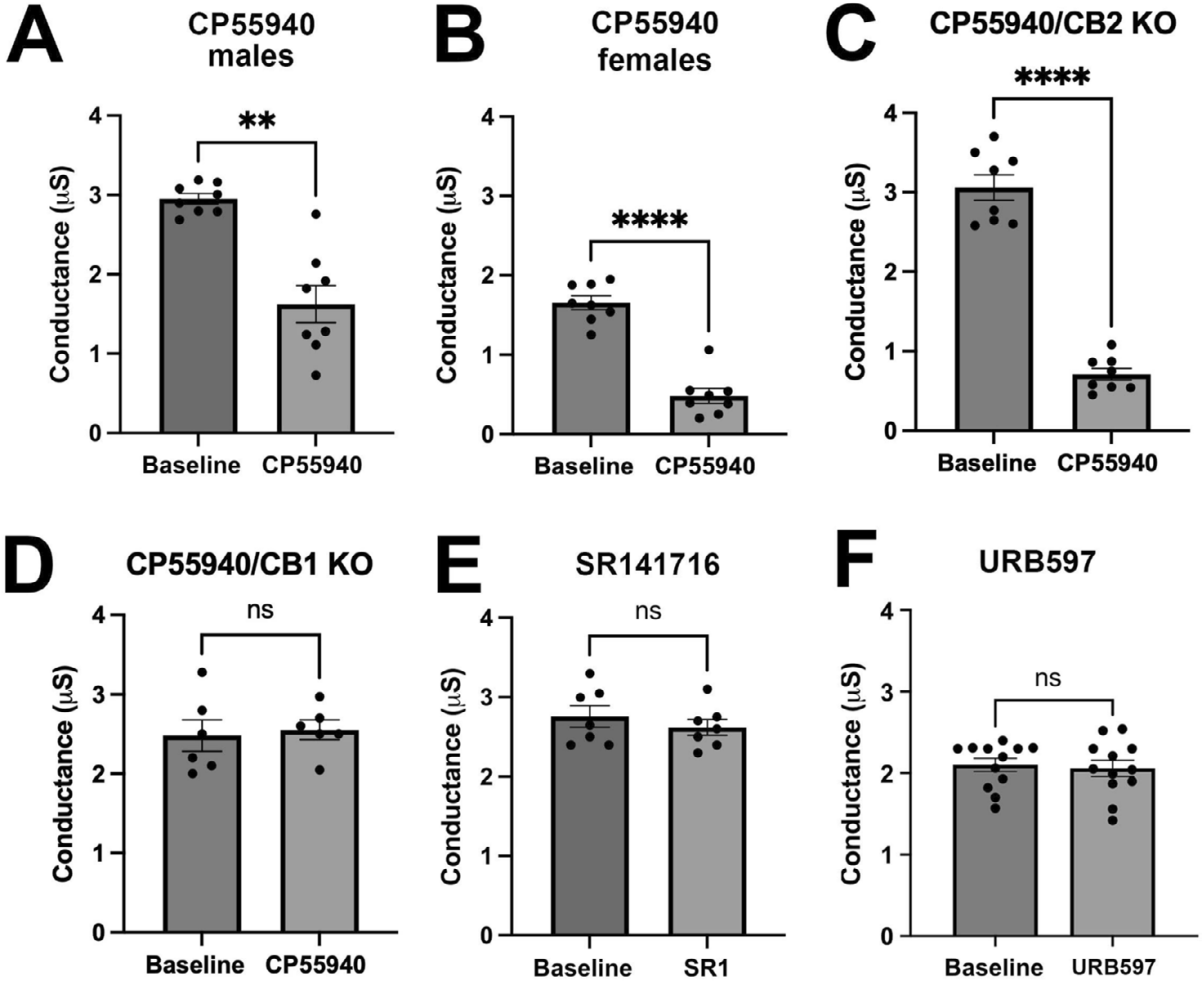
Cannabinoid receptor agonist effects on galvanic skin response. (A, B) Cannabinoid‐receptor agonist CP55940 (0.5 mg/kg, IP) reduces the galvanic skin response (GSR) in male (A) and female (B) mice. (C, D) CP55940 effects are seen in CB2 receptor but not CB1 receptor knockout mice. (E, F) CB1 receptor antagonist SR141716 (4 mg/kg, IP) and FAAH blocker URB597 (4 mg/kg, IP) each have no effect on GSR. **p < 0.01, ****p < 0.001 by paired *t*‐test vs. own baseline.

We next tested whether a cannabinoid receptor agonist would alter the galvanic skin response in mice. We found that the non‐selective agonist CP55940 (0.5 mg/kg, IP) substantially reduced the galvanic skin response in male mice an hour after treatment (Figure 3A, *p* = 0.002 by paired *t*‐test, *t* = 4.9, df = 7, *n* = 8). We separately tested a group of female mice under the same experimental conditions with a similar outcome (Figure 3B, *p* = 0.0001 by paired *t*‐test, *t* = 11.21, df = 7, *n* = 8).

We repeated the experiment in CB2 knockout mice (mixed male and female), finding that the CP55940 responses were intact (Figure 3C, *p* = 0.0001, *t* = 12.8, df = 7, *n* = 8), but in CB1 knockout mice, CP55940 was without effect (Figure 3D, *p* = 0.73, *t* = 0.36, df = 5, *n* = 6). The CB1 receptor antagonist SR141716 (4 mg/kg, IP) did not alter GSRs (Figure 3E, *p* = 0.40 by paired *t*‐test, *t* = 0.91, df = 6, *n* = 7). The FAAH blocker URB597 (4 mg/kg, IP) did not alter conductance (Figure 3F, *p* = 0.70 by paired *t*‐test, *t* = 0.40, df = 11, *n* = 12).

We also tested the CB2 agonist JWH133. Interestingly, at 4 mg/kg (IP), this drug also modestly reduced the galvanic skin response (Figure S1A, *p* = 0.0001, *t* = 6.2, df = 10, *n* = 11). However, because a similar effect was seen in CB2 knockouts (Figure S1B, *p* = 0.01, *t* = 3.0, df = 11, *n* = 12), the effect was not due to CB2 activation.

### 3.3 Phytocannabinoid Effects on Galvanic Skin Responses

**FIGURE 4:**
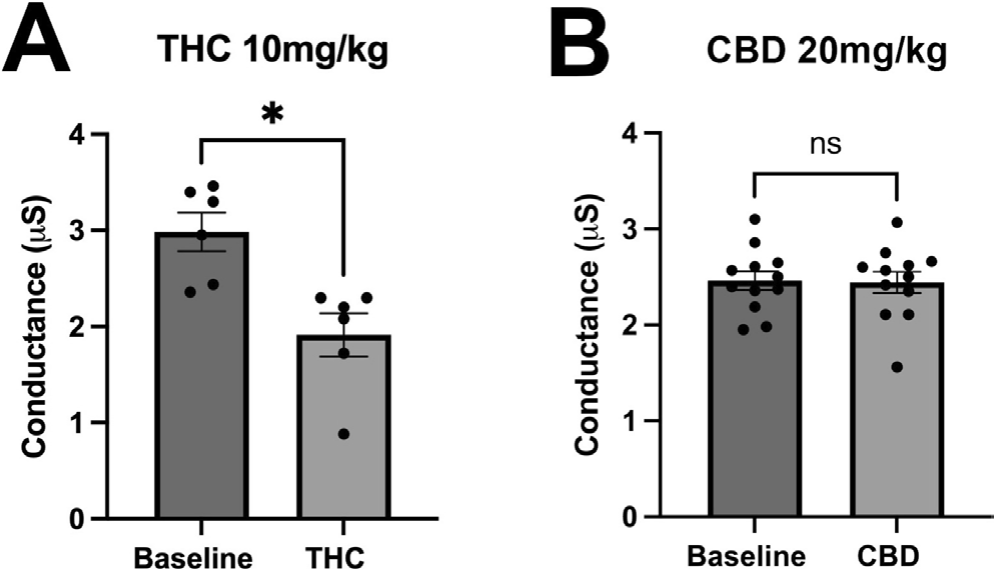
Phytocannabinoid effects on galvanic skin response. (A) THC (10 mg/kg, IP) lowers the galvanic skin response. (B) CBD (20 mg/kg, IP) does not. *p = 0.02, by paired *t*‐test.

We next tested whether Δ^9^‐tetrahydrocannabinol (THC) or cannabidiol (CBD) would affect the galvanic skin responses. We found that THC (10 mg/kg, IP) also reduced the GSR (Figure 4A, *p* = 0.02 by paired *t*‐test, *t* = 3.41, df = 5, *n* = 6). CBD did not have an effect at 20 mg/kg (Figure 4B, *p* = 0.90 by paired *t*‐test, *t* = 0.12, df = 11, *n* = 12).

### 3.4 Cannabinoids and Body Temperature

Cannabinoid receptor activation produces a minor reduction in body temperature in humans at physiological doses [27]. In principle, a drop in body temperature might account for some of the sweat reduction reported here. The synthetic cannabinoid JWH018 is a potent full agonist at the CB1 receptor [28] that has been shown to produce strong hypothermia in rodents (the effects at 0.5 mg/kg JWH018 are similar to 10 mg/kg of THC) [29]. We tested the effect of the synthetic cannabinoid JWH018 on sweating, while also monitoring body temperature. We found that JWH018 (0.5 mg/kg (IP)) lowered body temperature by 1.5 degrees Celsius (Data not shown: Baseline temperature (°C ± SEM): 38.3 ± 0.09; JWH018: 36.8 ± 0.31, *n* = 11; *p* = 0.0004 (*t* = 5.17, df = 10), *n* = 11 by paired *t*‐test vs. baseline). This concentration yielded only a slight reduction in baseline sweating (Baseline sweating (μS ± SEM) 2.14 ± 0.11; JWH018: 1.83 ± 0.14, *n* = 11; *p* = 0.014 (*t* = 2.95, df = 10) by paired *t*‐test vs. baseline). While statistically significant, this 14% drop is modest relative to the 36% reduction seen with THC.

### 3.5 CB1 Receptor Expression in the Walking Pad of the Mouse

**FIGURE 5:**
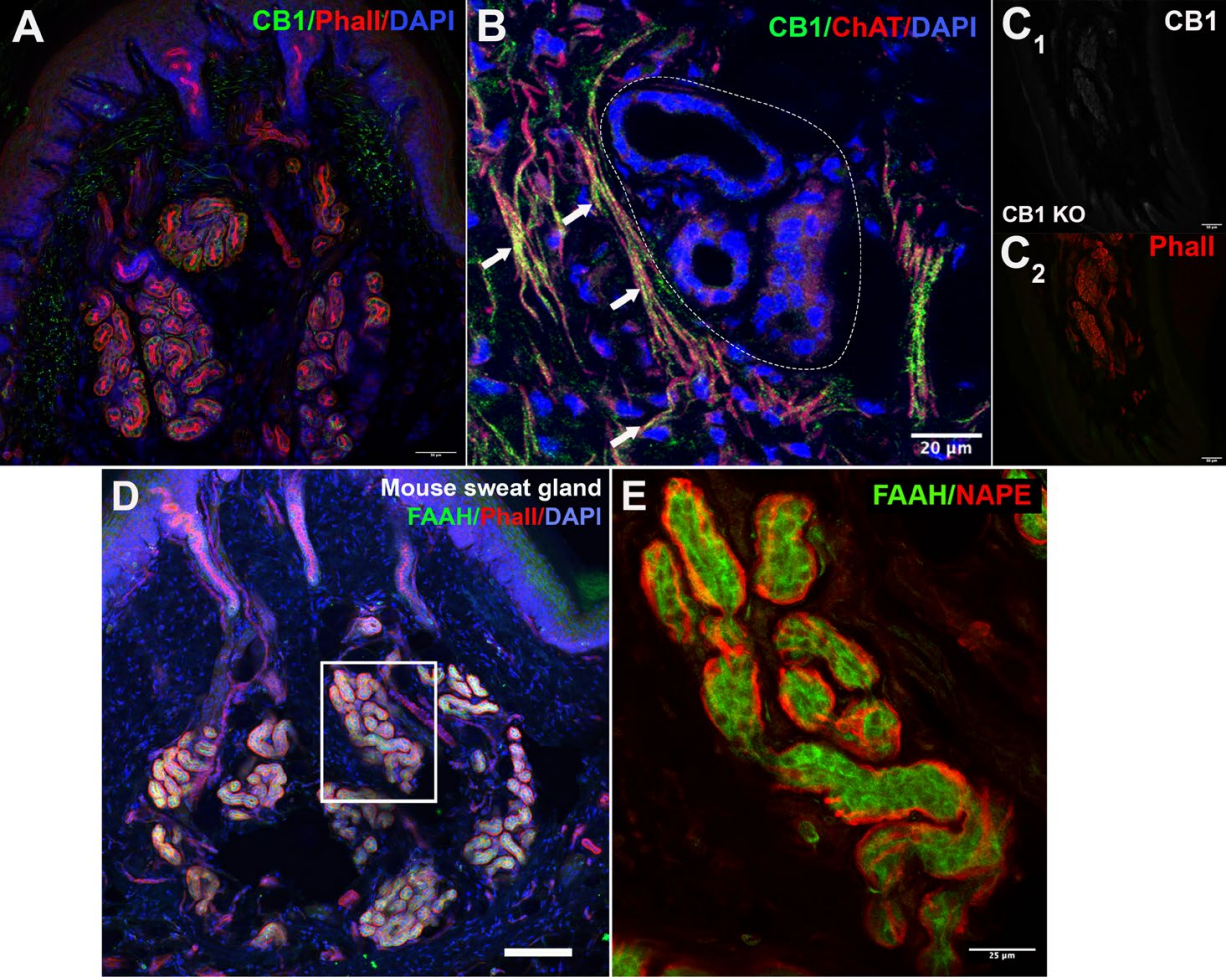
Cannabinoid‐related protein expression in the mouse walking pad. (A) The transverse section of the mouse footpad shows the coiled eccrine sweat glands as well as several ducts penetrating the epithelial layer. CB1 receptors (green) are seen chiefly in axon‐like processes around the glands. F‐Actin label Phalloidin (Phall, red) and nuclear marker DAPI (blue) are included as structural counterstains. (B) Arrows indicate that CB1 co‐stains with cholinergic marker choline acetyltransferase (ChAT) in axons adjoining the gland. (C) The upper panel shows CB1 staining in the CB1 knockout walking pad. The lower panel shows corresponding phalloidin (red) to identify sweat glands. (D) Staining for FAAH (green) appears in acinar cells. (E) The inset shows FAAH (green) and NAPE (red) staining. Scale bars: A, C–D = 50 μm, B = 20 μm, E = 25 μm.

Using immunohistochemistry, we tested for expression of CB1 receptors in the walking pad of the mouse. We observed axon‐like staining around the sweat glands (Figure 5A). This staining colocalized with the cholinergic marker choline acetyl transferase (ChAT, Figure 5B). Staining was absent in CB1 knockout paws (Figure 5C). We also observed protein expression for enzymes that synthesize and metabolize the endocannabinoid anandamide. The anandamide‐metabolizing enzyme fatty acid amide hydrolase (FAAH) is expressed within acinar cells (Figure 5D,E), whereas the anandamide‐synthesizing enzyme was seen in an expression pattern that likely corresponds to myoepithelial cells (Figure 5E).

## 4 Discussion

Cannabinoid CB1 receptors inhibit the function of several exocrine glands that are responsible for tearing and salivation [1, 2, 3], likely accounting for the dry mouth and eyes reported by cannabis users. Several lines of evidence, including an intriguing case study [16, 17], point to a cannabinoid effect on exocrine sweating, but since available rodent models have major limitations, we adapted a galvanic skin response‐based method to mice. We report that this model yields rapid, sensitive, and repeatable measures in awake, unanesthetized mice, with expected changes in response to muscarinic agonist and antagonist treatment. Our chief finding using this model is that a functional cannabinoid CB1 receptor signaling system reduces the skin conductance, consistent with an inhibition of eccrine sweating in these glands. Of the chief constituents of cannabis, THC reduces skin conductance while CBD has no effect. Lastly, CB1 receptors are expressed in axons adjoining the sweat glands, while anandamide‐synthesizing (NAPE‐PLD) and metabolizing (FAAH) enzymes are detected in sweat glands. This suggests that, as in lacrimal, submandibular, and parotid glands, there is a local CB1/anandamide‐based cannabinoid circuit in the walking pads of the mouse.

### 4.1 A Galvanic Skin Response Model of Sweating in Mice

Indirect and anecdotal lines of evidence suggest that cannabis may regulate sweating, but this has not been investigated systematically. In part, this may be because the main animal model of sweating relies on a starch‐iodine method to visualize secretion from sweat glands. The test is slow, cumbersome, and difficult to implement without anesthesia, a potential confound. Since the paws must, as a rule, be prevented from use for 10 min or more, this requires some form of restraint, itself a potential confound since sweating is directly affected by sympathetic activation, such as stress. Consistent photographic visualization of paws in terms of angle, distance, and focus can represent an additional challenge in an awake mouse. Most applications also require stimulation of sweating with pilocarpine, a limitation discussed below.

A staple of so‐called ‘lie detectors’, tests of the galvanic skin response have a long history of use in human subjects. When adapted to mice, with a hind paw positioned on each of the sensors, stable conductance readings were readily resolved well within the sensor range. The measures are stable and consistent both within‐group and over time, and we were able to validate the method by testing the muscarinic agonist pilocarpine—a standard means to induce sweating—finding that it increased the conductance. The peripherally restricted muscarinic antagonist glycopyrrolate, approved as a topical treatment to reduce sweating in humans, was effective in reducing conductance. We also confirmed that the test was able to resolve the previously reported age‐related reduction in sweating [26, 30]. We conclude that the assay of paw conductance is indeed a measure of sweating in mice.

The secretory regions of the mouse paw primarily reside in the central region of the paw, situated at the uppermost portion of the six walking pads (Figure 1A), i.e., the ‘business‐end’ of the paws that interact with the ground or, in this case, the sensor. Generally, we observed a rise in conductance over time, reaching a plateau over the course of 15–20 s (Figure 1C). We interpret the rise‐time to be due to the dryness of paws from walking on bedding. Bedding is presumably chosen for its absorbent properties and so leaves the paws relatively dry.

The structure of murine coiled ductile sweat glands, their cellular composition, and autonomic regulation all have much in common with humans [5]. Mice have already proven valuable in numerous studies of sweating (e.g., [31, 32, 33]). Sweating in mice is mediated by the release of acetylcholine and has been demonstrated to increase in response to heat [34]. Still, the mouse as a model of sweating will have limitations that should be identified and acknowledged. As noted, humans are unusual in their heavy reliance on sweating for temperature regulation. Mice rely on several means to reduce body temperature, including their tails for temperature regulation (reviewed in [35]); the eccrine sweating that occurs in their paws likely subserves other functions [5]. One interesting study found that sweating increased friction and so aided paw gripping [36]. It has also been proposed that paw secretory glands in rats release major urinary proteins [37] that allow them to communicate. Mice do not possess the same complement of glands, such as apocrine glands [5]. In humans, apocrine glands are found primarily in the armpits and groin, responding to stressful stimuli [38], and are of particular interest for treatment. While it is important to acknowledge their limitations, mice offer a valuable window into exocrine physiology as well as a rich trove of transgenic resources such as the knockouts used in this study.

### 4.2 How Does CB1 Activation Reduce Sweating?

It remains to be determined to what extent our findings in mice translate to humans. As noted, a recent case report described a salutary effect of cannabis use for an individual with hyperhidrosis [16]. But more generally, a cannabis‐dependent reduction in sweating may have been overlooked because cannabis users do not generally engage in intense physical activity. The one cannabinoid‐related trial in human subjects examined CBD and exercise, reporting no effects on sweating [39]. Our negative results for CBD in mice are in concordance with this finding.

CB1 receptor‐mediated inhibitory effects on lacrimal and two distinct salivary glands raise the possibility that the cannabinoid signaling system globally inhibits exocrine glands. Such a finding would be important for several reasons. There are many such glands throughout the body with essential physiological roles. Moreover, the regulation of these glands is generally not well understood, and patients who suffer from exocrine gland dysfunction generally have few options. This applies also to sweating (e.g., [40]). Most of the available therapies (reviewed in [6]) target the glands themselves, either by plugging the pores or inhibiting the response to cholinergic activation using a muscarinic antagonist such as glycopyrrolate [41, 42] or through surgical/mechanical means such as microwave irradiation [43]. In the mid‐20th century, formaldehyde was in use [44, 45], an alarming approach to anyone who has used formaldehyde to preserve tissue. The use of botulinum toxin, which inhibits neurotransmitter release when applied locally, is the exception insofar as it targets the neurons innervating the glands [9, 46]. Botulinum toxin has served admirably in multiple roles, but its potential systemic toxicity and molecular size severely constrain potential therapies. Our limited understanding of the neuronal regulation of sweating has impeded the development of therapeutics. The cannabinoid signaling system may offer an attractive therapeutic target since the cannabinoids in the CNS often act as feedback inhibitors, inhibiting neurotransmitter release (reviewed in [47]). Our anatomical findings point to CB1 receptors on axons innervating in the close proximity of the glands. Sweating is regulated by the autonomic nervous system, with postganglionic neurons releasing acetylcholine to stimulate eccrine sweat production (reviewed in [48]). One model of CB1 inhibition of sweating would therefore be the inhibition of acetylcholine release due to CB1 expression on cholinergic inputs that weave through the glands in mice [30]. This mirrors what we have seen in other exocrine glands thus far, and we consider this to be the likely explanation. In the case of sweating, a second indirect mechanism may be in play: Hypothermia is a known consequence of cannabis use (e.g., [49]), and early studies after the identification of the CB1 receptor [11] implicated this receptor in hypothermia [50]. We made use of JWH018, a synthetic cannabinoid that is a potent full agonist at CB1 receptors and has been shown to produce a pronounced reduction in temperature relative to THC [29]. At a dose that produces temperature reductions comparable to 10 mg/kg of THC, JWH018 produced only a modest, albeit statistically significant, drop in baseline sweat responses. We consider it unlikely that reduced peripheral body temperature is the primary cause of the observed reduction in sweating with cannabinoid receptor agonists.

### 4.3 An Anandamide‐Based Circuit?

The cannabinoid signaling system makes use of two endogenous lipid messengers, anandamide and 2‐AG. In principle, either or both messengers could mediate effects on the sweat gland‐associated CB1 receptors. And while they may be synthesized locally, it is also possible that they arrive through some other pathway, such as the bloodstream. We find that the expression pattern of cannabinoid‐related proteins mirrors what we have seen in other tubuloacinar glands, such as the submandibular glands [2]. In general, tubuloacinar glands are made up of acinar cells that secrete the contents (e.g., sweat/saliva/tears) of a given gland. The acini are surrounded by myoepithelial cells with contractile properties to squeeze the acini and so promote secretion through ducts that are lined with ductal epithelial cells. In sweat glands, we see CB1 in cholinergic axons innervating the glands and NAPE‐PLD in myoepithelial cells, while FAAH is abundantly expressed in acinar cells. A potential circuit involves myoepithelial cell activity of NAPE‐PLD to produce anandamide that then acts on CB1 receptors in cholinergic axons. Excess anandamide is then metabolized by abundant FAAH in acinar cells, likely serving as a sump. This arrangement mirrors what we have reported in the submandibular salivary gland [2]. What stimulates NAPE‐PLD in myoepithelial cells is yet unclear. It is likely that in mice, as in the rat [21], sweating is mediated by activation of muscarinic M3 receptors. It is possible that NAPE‐PLD can be activated by G_q_‐coupled M3 receptors. Such an arrangement has been demonstrated for 2‐AG production in the CNS (e.g., [51]) and has been proposed for anandamide [52]. We tested whether blocking FAAH might reduce sweating by enhancing anandamide levels, but saw no effect. It is possible that there is insufficient anandamide tone under the experimental conditions to induce a response.

## 5 Conclusion

In summary, we have made use of a galvanic skin response‐based assay to measure the conductivity in the hind paws of awake, unanesthetized mice as a measure of sweating. We find the galvanic skin responses to be stable and consistent over time and, importantly, to be responsive to stimuli that increase or decrease basal sweating. Using this model, we determined that cannabinoid CB1 receptor activation reduces the galvanic skin response. We propose that cannabinoid CB1 receptor activation reduces basal sweating in mice. This effect may point the way to a new class of therapeutics for hyperhidrosis.

## Author Contributions

A.S. and N.M. conceived of the project, designed the experiments, collected data, analyzed data, prepared figures, and wrote the manuscript. J.Y. assisted in collecting data and reviewed the manuscript.

## Funding

The authors have nothing to report.

## Conflicts of Interest

The authors declare no conflicts of interest.

## Supporting information


**Figure S1:** CB2 agonist JWH133 causes a CB2‐independent reduction in galvanic skin response. (A) JWH133 treatment (4 mg/kg, IP) reduces the galvanic skin response 1 h after injection. (B) JWH133 similarly reduces the galvanic skin response in CB2 knockout mice. ***p = 0.0001, *n* = 11; *, *p* = 0.01, *n* = 12.

## Data Availability

Data for Figures 2, 3, 4 will be available in a file accompanying the manuscript and also on request from the corresponding author.
