# Supplementary figures and images for: Don't Sweat It: Cannabinoid CB1 Receptors Reduce Sweating in a Mouse Model
Source: FASEB J. 2026 Jun 13;40(12):e72051. doi: 10.1096/fj.202601143R (PMC13264398; doi:10.1096/fj.202601143R)

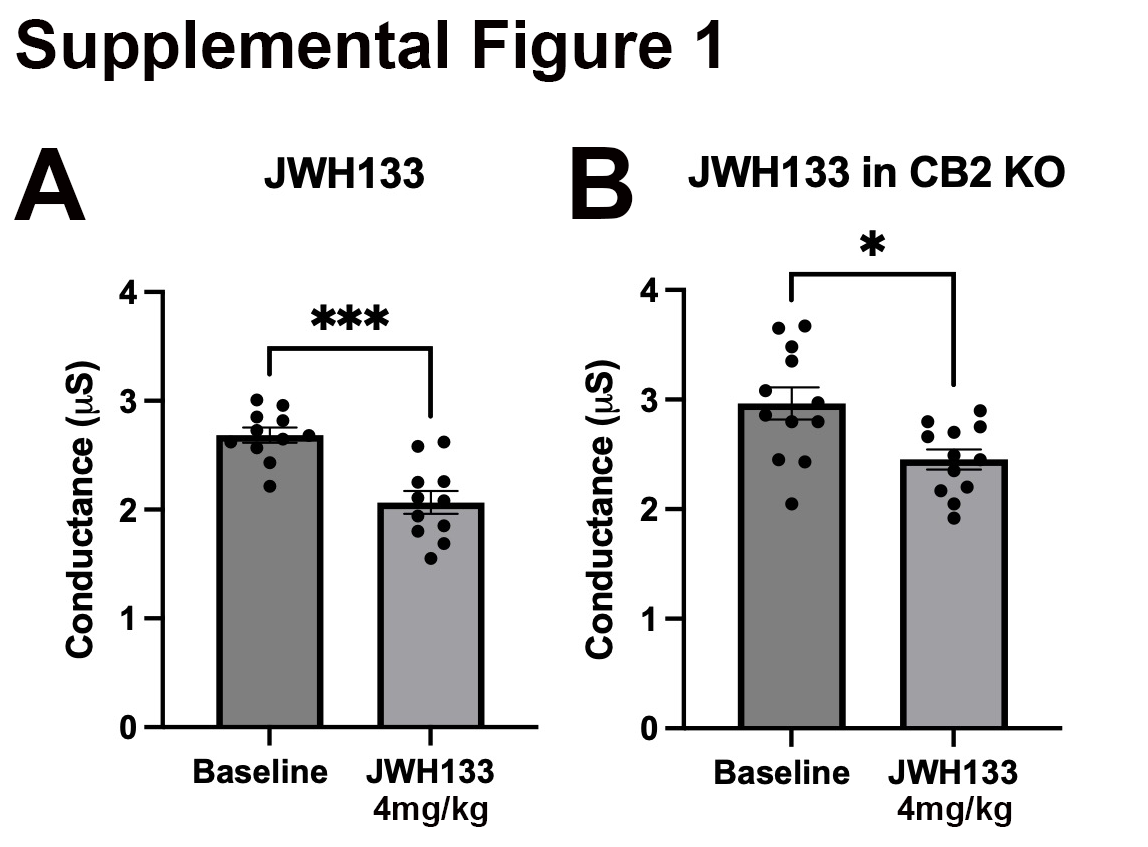

Supplement: Supplementary file 1 — Figure S1: CB2 agonist JWH133 causes a CB2‐independent reduction in galvanic skin response. (A) JWH133 treatment (4 mg/kg, IP) reduces the galvanic skin response 1 h after injection. (B) JWH133 similarly reduces the galvanic skin response in CB2 knockout mice. ***p = 0.0001, n = 11; *, p = 0.01, n = 12. [file FSB2-40-e72051-s001.tif]
